# Heterotrophic respiration does not acclimate to continuous warming in a subtropical forest

**DOI:** 10.1038/srep21561

**Published:** 2016-02-22

**Authors:** Chuansheng Wu, Naishen Liang, Liqing Sha, Xingliang Xu, Yiping Zhang, Huazheng Lu, Liang Song, Qinghai Song, Youneng Xie

**Affiliations:** 1 Key laboratory of Tropical Forest Ecology, Xishuangbanna Tropical Botanical Garden, Chinese Academy of Sciences, Mengla, 666303, China; 2 Ailaoshan Station for Subtropical Forest Ecosystem Studies, Jingdong, 676209, China; 3 University of Chinese Academy of Sciences, Beijing, 100049, China; 4 Global Carbon Cycle Research Section, Center for Global Environmental Research, National Institute for Environmental Studies, Tsukuba, 305-8506, Japan; 5 Key laboratory of Ecosystem Network Observation and Modelling, Institute of Geographic Sciences and Natural Resources Research, Chinese Academy of Sciences, Beijing, 100101, China; 6 Jingdong Bureau of National Nature Reserve, Jingdong, Yunnan 676209, China

## Abstract

As heterotrophic respiration (*R*_H_) has great potential to increase atmospheric CO_2_ concentrations, it is important to understand warming effects on *R*_H_ for a better prediction of carbon–climate feedbacks. However, it remains unclear how *R*_H_ responds to warming in subtropical forests. Here, we carried out trenching alone and trenching with warming treatments to test the climate warming effect on *R*_H_ in a subtropical forest in southwestern China. During the measurement period, warming increased annual soil temperature by 2.1 °C, and increased annual mean *R*_H_ by 22.9%. Warming effect on soil temperature (*WE*_T_) showed very similar pattern with warming effect on *R*_H_ (*WE*_RH_), decreasing yearly. Regression analyses suggest that *WE*_RH_ was controlled by *WE*_T_ and also regulated by the soil water content. These results showed that the decrease of *WE*_RH_ was not caused by acclimation to the warmer temperature, but was instead due to decrease of *WE*_T_. We therefore suggest that global warming will accelerate soil carbon efflux to the atmosphere, regulated by the change in soil water content in subtropical forests.

Global soil CO_2_ efflux was estimated to be 80.4 Pg C yr^−1^ from 1980 to 1994^1^, a value that had increased to 98.0 Pg C yr^−1^ by 2008^2^. Global soil organic carbon was estimated to be about 3300 Pg, four times the amount present in living plants globally^3^,^4^. There is a positive relationship between global soil CO_2_ efflux and temperature with an increase of 3.3 Pg C yr^−1^ per °C^1^, indicating that increased temperature has a great potential to affect soil CO_2_ efflux and produce a positive carbon–climate feedback under global warming. Forests cover only about 30% of the terrestrial ecosystems, but contain about 45% of terrestrial carbon^5^. Therefore, forest soil organic carbon decomposition plays an important role in the regional and global carbon cycle and studies have increasingly been conducted to explore the effects of warming on forest soil organic carbon decomposition, aiming to improve prediction of carbon–climate feedbacks.

Many previous studies focused on the warming effects on soil respiration^6^,^7^,^8^,^9^,^10^. However, soil respiration includes two main components: heterotrophic respiration (*R*_H_) and autotrophic respiration (*R*_A_). Responses of heterotrophic respiration (*R*_H_) and autotrophic respiration (*R*_A_) to warming are not always the same. For example, Schindlbacher, *et al.*^11^ reported that *R*_H_ and *R*_A_ responded similarly to temperature increase; both increasing with warming. However, Zhou, *et al.*^12^ reported a negative effect of warming on both *R*_H_ and *R*_A_. Other studies showed that warming increased *R*_H_, but decreased *R*_A_^13^,^14^. Because only the *R*_H_ component of soil respiration has potential to increase atmospheric CO_2_^15^, it is important to separate *R*_H_ from soil respiration and to examine how warming may affect *R*_H_^16^,^17^,^18^.

Forest soil organic carbon decomposition plays an important role in the carbon cycle, but most previous studies about *WE*_RH_ were carried out in grassland ecosystems^12^,^13^,^14^,^19^,^20^,^21^. A few studies have been conducted in boreal coniferous forest dominated by Norway spruce^11^,^22^,^23^ and in a temperate forest^18^. To our knowledge, no studies have been reported in subtropical forests.

The subtropical evergreen broad-leaved forest in the Ailao Mountains in Yunnan of southwestern China has been considered a carbon sink^24^, but the carbon sink strength is likely to be weakened by warming^25^. During the period from 1961 to 2004, the mean annual air temperature over the Yunnan Plateau (southwestern China) increased at a rate of 0.3 °C per decade^26^. A recent study showed that the mean annual air temperature increased by 0.36 °C per decade from 1983 to 2010, leading to an increasing trend of 0.31 °C per decade from 1986 to 2010 in the top 10 cm of soil in the subtropical forest^27^. Previous studies have shown that *R*_H_ exhibited seasonal variation and had a significant positive relationship with soil temperature^28^,^29^. However, it remains unclear how soil warming affects *R*_H_ in this subtropical forest.

Previous studies have showed inconsistent results about *WE*_RH_. A few studies suggested that warming decreased *R*_H_^12^,^20^, while some demonstrated a positive effect on *R*_H_ which can be sustained for 5–10 years^18^,^30^,^31^. Other studies argued acclimatization in terms of the warming effect declining over time^8^,^22^,^32^, which may be ascribed to depletion of soil labile carbon^16^,^19^,^33^,^34^ or acclimatization of soil microbes^35^,^36^,^37^,^38^. Other studies also argued acclimatization in terms of temperature sensitivity adaption^11^,^17^,^21^. Based on mentioned studies above, we hypothesized that *R*_H_ acclimated to continuous warming in this forest. To test this hypothesis, we conducted ‘trenching alone’ and ‘trenching with warming’ treatments in this forest.

## Results

### Treatment effects on soil environmental factors and soil carbon efflux

**Figure 1:**
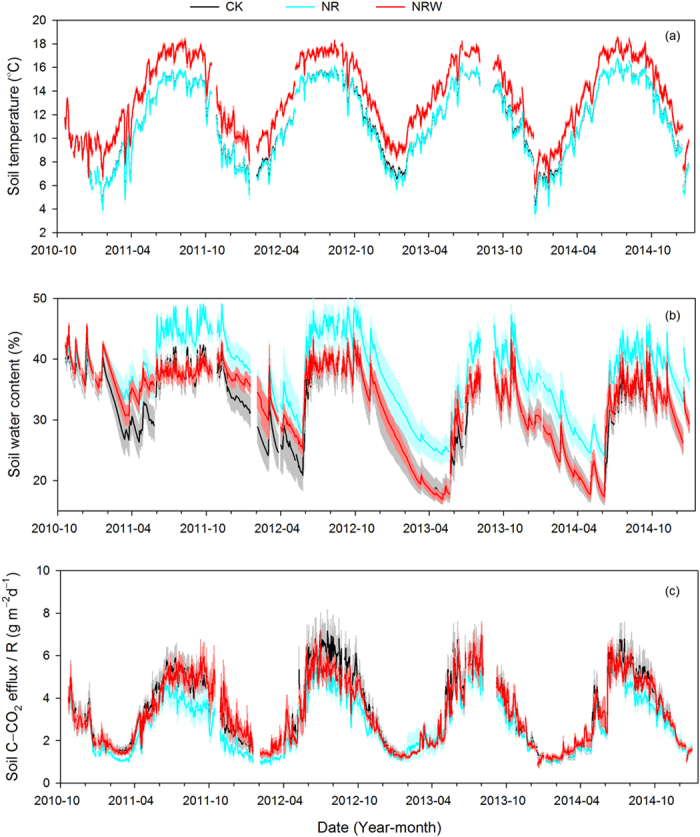
Seasonal variations of soil temperature (**a**), soil water content (**b**), and soil carbon efflux (**c**) after treatments began. Black lines with grey shadows represent the control treatment (CK), cyan lines with semi-transparent cyan shadows represent the trenching treatment (NR), and red lines with semi-transparent red shadows represent the trenching together with warming treatment (NRW) (Mean ± SE).

Compared with the control treatment (CK), the trenching treatment (NR) did not change soil temperature (*T*, °C) (Fig. 1a), but increased soil water content (*W*, % (v/v)) (Fig. 1b). The trenching together with warming treatment (NRW) increased *T* (Fig. 1a), but did not change *W*, except for the first 1.5 years (Fig. 1b). Soil carbon efflux reduced in the NR treatment in the first year and changed little in the later years during the measurement period. In the NRW treatment, soil carbon efflux changed little (Fig. 1c).

Compared with the NR treatment, the NRW treatment increased *T*, but decreased *W* (by annual average of 5.1%, v/v) (Fig. 1a,b) and increased *R*_H_ especially in the first year (Fig. 1c).

**Figure 2:**
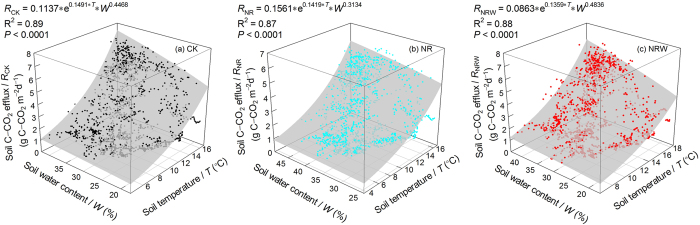
Two-factor regression of soil carbon efflux with soil temperature and soil water content.

There were similar seasonal variations in *R*, *T*, and *W* of the CK, NR, and NRW treatments (Fig. 1). The Pearson correlation analysis showed *R* more correlated to *T* (Table S1), and the two-factor regression model showed that *R* had a positive relationship with both *T* and *W* (Fig. 2).

### Warming effects on *R*_H_ and *T*

To clarify the effect of warming on *R*_H_, we focus on the affecting factor of changes to soil water content. Two-factor regression models (Fig. 2) were used for correction (for details see the data analysis section).

**Figure 3:**
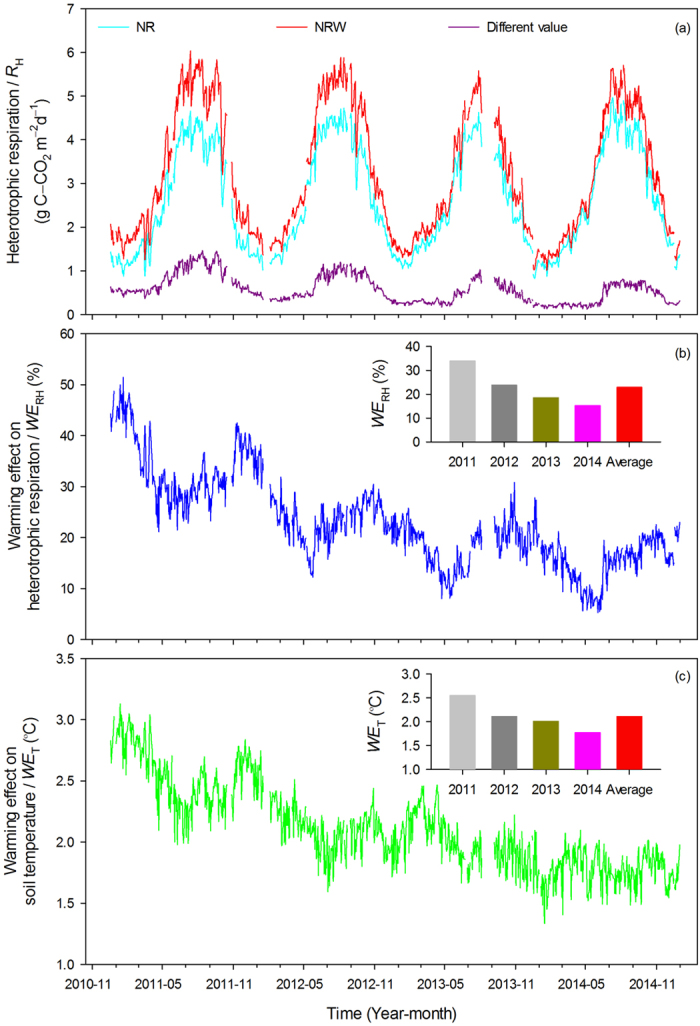
Variation of heterotrophic respiration in warmed (NRW) and unwarmed (NR) treatments and their difference (*R*_NRW_ − *R*_NR_) (**a**), the variation of the warming effect on heterotrophic respiration (*WE*_RH_) (**b**), and the variation of the warming effect on soil temperature (*WE*_T_) (**c**).

Results show that *R*_H_ showed seasonal variation in both the NR and NRW treatments, with maximum values appearing in July or August and minimum values occurring in February. The difference value between NR and NRW (*R*_NRW_ − *R*_NR_) also exhibited seasonal variation (Fig. 3a). *WE*_RH_ had similar seasonal variation to *R*_H_ with about 3 months delay; during the measurement period, *WE*_RH_ was 33.9, 23.8, 18.5, and 15.3% for 2011–2014, respectively (with an average of 22.9%), clearly decreasing year by year (Fig. 3b). Variation of the warming effect on *T* (*WE*_T_) was very similar to that of *WE*_RH_, and *WE*_T_ was 2.6, 2.1, 2.0, and 1.8 °C (with an average of 2.1 °C) for 2011–2014, respectively (Fig. 3c).

### Factors affecting the warming effect on *R*_H_

**Figure 4:**
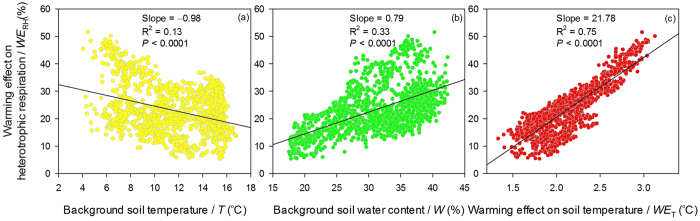
Relationships between *WE*_RH_ and *T* (**a**), *W* (**b**), and *WE*_T_ (**c**).

The factors *T*, *W*, and *WE*_T_ had a significant relationship with *WE*_RH_ (*p* < 0.0001), which meant *WE*_RH_ was affected by *T*, *W*, and *WE*_T_. The factors *T*, *W*, and *WE*_T_ explained 13, 33, and 75% of *WE*_RH_’s variation, respectively. A 1 °C increase in *T* and a 1% decrease in *W* would reduce 0.98 and 0.79% of *WE*_RH_, respectively; however, a 1 °C increase in *WE*_T_ would cause a 21.78% increase in *WE*_RH_ (Fig. 4). This suggests that a range of increased soil temperature (*WE*_T_) control *WE*_RH_.

## Discussion

Previous studies have shown that *R*_H_ acclimated to warmer temperature over the warming period^8^,^22^,^32^. However, several long-term warming experiments have shown sustained *WE*_RH_ in ecosystems with high soil organic carbon^18^,^30^,^31^. This indicates that such sustained stimulation may be attributed to high substrate availability^18^. In this study, soil organic carbon, soil labile organic carbon, and soil microbial carbon did not significantly (*p* > 0.05) decrease in NR and NRW comparing to CK during the 4-year measurement period, but they were larger in NRW than that in NR (Fig. S2). In this case, during the 4-year measurement period, substrate is unlikely to be a limiting factor^28^,^39^. Our results show that *WE*_RH_ declined year by year. Without further analyses, this indicated “acclimatization” superficially (Fig. 3b). However, this decline probably was not caused by acclimation but was caused by the reduction in *WE*_T_ over the 4-year observation period (Figs 3c and 4c). The temperature sensitivity (*Q*_10_) analysis showed that warming increased *Q*_10_ in the later period of this study which showed opposite result to previous studies^11^,^17^,^21^ and indicated the absence of “acclimatization” (Fig. S3). A current meta-analysis showed that *WE*_RH_ remained stable during warming period, which also challenged “acclimatization” of microbial activity to warmer temperature^40^. Therefore, *WE*_T_ is likely to be the controlling factor in this study.

In this study, *WE*_RH_ (22.9%) was lower than that of Aguilos, *et al.*^18^, Schindlbacher, *et al.*^11^ and Eliasson, *et al.*^22^ reported (82.0%, 42.0% and >30%, respectively). *WE*_T_ values in the experiments of Aguilos, *et al.*^18^, Schindlbacher, *et al.*^11^ and Eliasson, *et al.*^22^ were 3, 4, and 5 °C, respectively, all larger than ours (2.1 °C). A review showed that warming by 2.0 °C increased *R*_H_ by an average of 21.0%^40^, similar to our result. Another 2-year soil monolith transplantation experiment at the same site demonstrated that soil organic carbon efflux increased by 70.5 and 62.6% because of an annual *WE*_T_ of 3.9 and 6.7 °C (unpublished data, Fig. S4). These results suggest that a positive relationship between *WE*_RH_ and *WE*_T_ requires a specific range of *WE*_T_, and when *WE*_T_ exceeds this range, *WE*_RH_ becomes restricted (Fig. S4). Therefore, *WE*_T_ controlled *WE*_RH_ in this subtropical forest.

Although soil temperature is more important than soil water content on affecting dynamics of soil carbon efflux (Fig. 1, Table S1), dynamics of soil water content may also affect *WE*_RH_. For example, Liu, *et al.*^20^ found that warming decreased soil water content, which resulted in a reduction in *R*_H_. Similarly, a prolonged summer drought offsets soil warming effects^9^. Our results also suggest that soil water content affected *WE*_RH_, mediating *WE*_RH_ under the warmer soil temperature conditions (Fig. 4b).

In this study, *WE*_T_ decreased yearly, perhaps owing to aging of warming lamps. *WE*_T_ also showed seasonal variation (Fig. 3c). These factors should be considered in future research when researchers are attempting to maintain a constant *WE*_T_. Nevertheless, these results allowed us to analyse the *WE*_T_ effect on *WE*_RH_. In this forest, *R*_H_ is 9.53 t C ha^−1^ yr^−1^ (Fig. 3a), and given an increase of 1 °C in *WE*_T_, *WE*_RH_ will increase by 21.78%, an additional soil carbon release to the atmosphere can be estimated to be about 2.65 t ha^−1^ yr^−1^ from the subtropical forest.

Response of soil organic carbon to temperature depends on soil temperature^41^ and quality of soil organic carbon^42^,^43^. Factors affecting soil carbon quality and decomposition processes can influence the temperature response and thus warming effect^44^. As soil water also affects soil carbon efflux, it’s changes should be also considered to prediction of carbon–climate feedbacks^40^,^45^. Further studies should focus on responses of *WE*_RH_ and interactions between factors.

## Conclusions

In summary, our warming experiment increased soil temperature by 2.1 °C and thereby increased heterotrophic respiration by 22.9% during the 4-year observation period. Continuous measurement allowed us to analyse variations of *T*, *W*, *WE*_T_, and *WE*_RH_ and their inter-relationships. Our results show that the warming effect on *R*_H_ was controlled by a range of increased soil temperature and regulated by the variation of soil water content. This suggests that global warming will accelerate soil carbon efflux to the atmosphere, regulated by a change of soil water content in response to warming and rainfall changes. Future warming in subtropical forests can accelerate release of soil organic carbon to the atmosphere.

## Materials and Methods

### Site description

This experiment was conducted at the Ailaoshan Station for Subtropical Forest Ecosystem Studies (24°32′N, 101°01′E; 2480 m above sea level) of the Chinese Ecological Research Network, which is located in Jingdong County, Yunnan Province, China. The annual mean air temperature was 11.3 °C, with a minimum monthly mean temperature of 5.7 °C in January and a maximum monthly mean temperature of 15.6 °C in July. Average annual rainfall was 1778 mm, with 86.0% of this falling in the rainy season (May–October)^28^. The dominant tree species in the forest are *Lithocarpus xylocarpus*, *Lithocarpus hancei*, and *Castanopsis wattii*. The soils are Alfisols with a pH of 4.5, soil organic carbon of 304 g kg^−1^, and total nitrogen of 18 g kg^−1^ in the humus horizon^39^.

### Data collection

A multichannel automated chamber system for continuous measurement of soil CO_2_ effluxes was established in October 2010. The system comprised 20 automatic chambers (90 × 90 × 50 cm) and a control box (Fig. S1). On 17 December 2010, the 20 chambers were divided into four treatments (five chambers per treatment): control (CK), litter removal (NL), trenching (NR), and infrared light warming together with trenching (NRW). In this study, we only discuss the NR and NRW treatments and the control CK. The infrared light warming method has been frequently applied for soil warming in forest ecosystems^3^,^34^,^46^,^47^, and is also used in this study. The key advantages of this method are that there is no disturbance to the soil structure and it involves the same process of warming as heating from the climatic warming effect (heating soil from the surface to a deeper depth). For trenching treatments, a 1 m × 1 m square trench (width 30 cm, depth 50 cm) was dug to form a cube of soil contained by PVC planks; soil was backfilled by its original layers with topsoil over subsoil. The main components of the control box are an infrared gas analyser (Li-820, Li-Cor Inc., Lincoln, NE, USA) and a datalogger (CR1000, Campbell Scientific Inc., Logan, UT, USA), as described in more detail in earlier studies^48^,^49^.

Soil temperature (*T*, °C) at a 5-cm depth and air temperature (*T*a, °C) were measured inside each chamber using self-made thermocouples^49^. Soil water content (*W*, % (v/v)) at a 10-cm depth was monitored using time domain reflectometry (CS-616, Campbell Scientific Inc.)^49^. Air pressure (*P*, hPa) at a 30-cm height in the centre of the plot was measured by a pressure transducer (PX2760, Omega Engineering, Inc., Stamford, CT, USA)^49^.

Soil C–CO_2_ efflux (*R*) was calculated as follows:


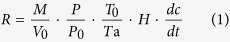


where *R* is soil C–CO_2_ efflux (g C–CO_2_ m^−2^ d^−1^); *M* is the C molar mass; *V*_0_, *P*_0_ and *T*_0_ are constants (22.4 L mol^−1^, 1013.25 hPa, and 273.15 K, respectively); *T*a and *P* are air temperature (K) and pressure (hPa), respectively; *H* the is height of the respiration chamber (m); and *dc/dt* is the slope of CO_2_ concentration variation with time over the measurement period (measurement for 3 min and the calculation ignores data from the first minute).

### Data analysis

Every day, each chamber received 24 data points for *R* (hourly), and 48 data points for *T* and *W* (twice per hour), except during periods of electrical failure. For each chamber, available data were used to calculate daily average values. Previous analysis showed that *R*_H_ was unusual in the fifth group for both NR and NRW (Fig. S1); therefore, the fifth group was ignored, leaving four groups as the four repeats in this study.

The two-factor regression model was used to quantify the relationship of soil temperature (*T*) and soil water content (*W*) with soil carbon efflux (*R*) as follows^50^:


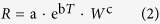


where a, b, and c are constants estimated from the regression model by the nonlinear regression dynamic fit wizard using Sigmaplot (Version 12.5, Systat. Software, Inc., Point Richmond, CA, USA) (for details see Fig. 2).

Figure 2 suggests that *T* and *W* had a positive effect on *R*, while treatments changed the soil microclimate (Fig. 1a,b). Compared to CK, NR increased soil water content; however compared to NR, NRW decreased soil water content (Fig. 1b). To eliminate the biases due to changed soil water content, *WE*_RH_ should be calculated under the same soil water content condition. Therefore, the background soil temperature and soil water content measured in the control treatment were used to correct the values of *R*_NR_, using Equation (2). For NRW, the soil temperature of the NRW plots and the background soil water content of the control plots were used for the correction, so that the warming effect was due to the soil temperature increase without the effect of soil water content decrease (Fig. 3b). Parameters (a, b, and c) for NR and NRW were shown in Fig. 2b,c, and their estimation results were shown in Table S2.

Warming effects on *R*_H_ (*WE*_RH_, %) and on soil temperature (*WE*_T_, °C) were calculated using the following equations:


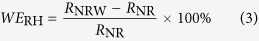



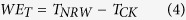


## Additional Information

**How to cite this article**: Wu, C. *et al.* Heterotrophic respiration does not acclimate to continuous warming in a subtropical forest. *Sci. Rep.* **6**, 21561; doi: 10.1038/srep21561 (2016).

## Supplementary Material

Supplementary Information
