# Supplementary material for: Heterotrophic respiration does not acclimate to continuous warming in a subtropical forest
Source: Sci Rep. 2016 Feb 22;6:21561. doi: 10.1038/srep21561 (PMC4761939; doi:10.1038/srep21561)
Supplement: Supplementary Information [file srep21561-s1.pdf]

Does heterotrophic respiration acclimate to continuous warming in a subtropical forest?

Chuansheng Wu[1, 2, 3], Naishen Liang[4], Liqing Sha[1, 2], Xingliang Xu[5], Yiping Zhang[1, 2*], Huazheng Lu[1,3], Liang Song[1], Qinghai Song[1], Youneng Xie[6]

[1]Key laboratory of Tropical Forest Ecology, Xishuangbanna Tropical Botanical Garden, Chinese Academy of Sciences, Mengla, 666303, China, [2]Ailaoshan Station for Subtropical Forest Ecosystem Studies, Jingdong, 676209, China, [3]University of Chinese Academy of Sciences, Beijing, 100049, China, [4]Global Carbon Cycle Research Section, Center for Global Environmental Research, National Institute for Environmental Studies, Tsukuba, 305-8506, Japan, [5]Key laboratory of Ecosystem Network Observation and Modelling, Institute of Geographic Sciences and Natural Resources Research, Chinese Academy of Sciences, Beijing, 100101, China, [6]Jingdong Bureau of National Nature Reserve, Jingdong, Yunnan 676209, China

* Correspondence and requests for materials should be addressed to Y.Z. (yipingzh@xtbg.ac.cn)

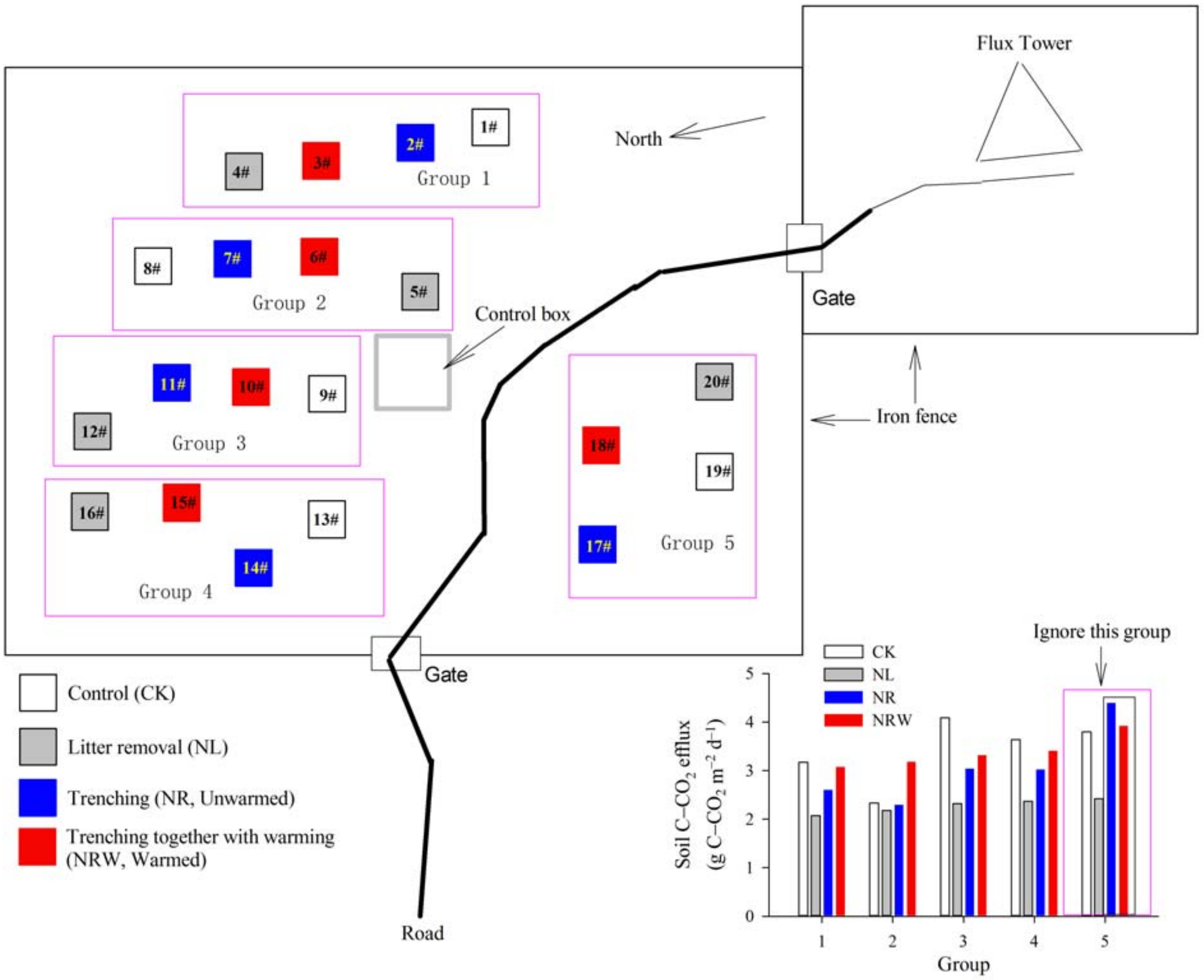

Fig. S1 Sample plot figure and treatment positions. Previous analysis (from our original data) provided for the annual efflux mean of each subplot.

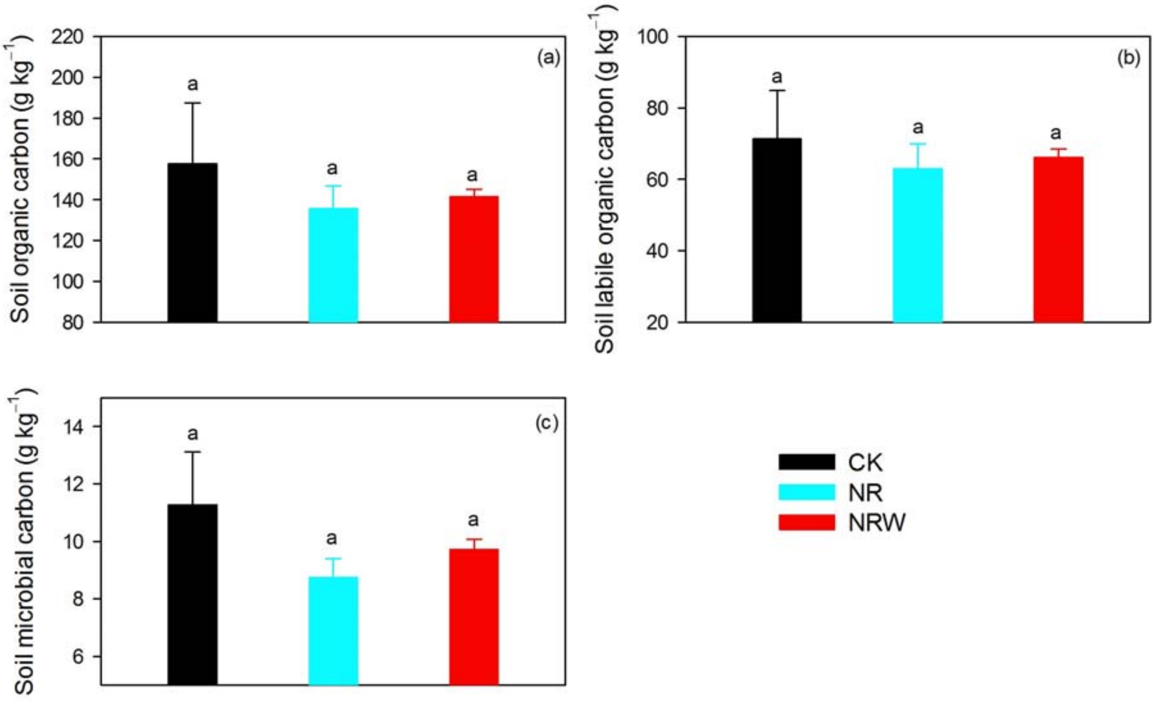

Fig. S2 Comparisons of soil organic carbon (SOC) (a), soil labile organic carbon (LOC) (b), and soil microbial carbon (MBC) (c) among treatments. Data are mean ± SE. Soil samples were collected in September 2014. All data passed the normality, but failed homoscedasticity tests. Therefore we conducted one-way ANOVA analysis with Tamhane's post-hoc test. Same letters indicate no significant differences.

Methods for measuring MBC, SOC and LOC

We collected soil samples at 0-10 cm and then divided each soil sample into two parts. One part was immediately removed roots and gravel and then passed through a 2 mm sieve. Sieved soil samples were stored in a cooling box and sent to the Biogeochemistry Laboratory of the Xishuangbanna Tropical Botanical Garden for measuring soil microbial carbon (MBC). The other part was dried by air in the laboratory of Ailaoshan Station for Subtropical Forest Ecosystem Studies. After removing roots and gravel, the air-dried soil samples were sent to the Biogeochemistry Laboratory of the Xishuangbanna Tropical Botanical Garden for measuring soil organic carbon (SOC) and soil labile

organic carbon (LOC).

MBC was measured by chloroform fumigation–extraction method (Wu et al., 1990) as follows:

1. Fumigating a portion of each soil sample (7 ± 0.01 g) with ethanol-free chloroform for 24 h at 25 °C in a sealed incubator in the dark. Another portion of each soil sample (7 ± 0.01 g) was not fumigated.
2. Removing the chloroform completely from the soils by air exhaust to enhance evaporation.
3. Extracting the fumigated and unfumigated samples with 35 ml of freshly prepared 0.05 mol L^{-1} K_2SO_4 by capping and shaking at 300 rpm for 1 h.
4. Centrifugation of the suspensions for 10 min at 5000 rpm and filtering the supernatants through 0.45 μm nitrocellulose membrane filters (Pall Life Science Company, Beijing, China).
5. Analyzing these filtered samples for Dissolved Organic Carbon (DOC) using a total organic carbon (TOC) / total nitrogen (TN) analyzer (LiquiTOC II, Elementar Analyzer System, Germany).
6. DOC was determined on the unfumigated filters, and the difference in DOC between the unfumigated and fumigated filters was MBC.

SOC and LOC were measured in soil oxidized with $K_2Cr_2O_7$-H_2SO_4 at the Biogeochemistry Lab at the Xishuangbanna Tropical Botanical Garden (Hu et al., 2012). We used a solution of 0.8 mol L^{-1} 1/6$K_2Cr_2O_7$ mixed 1:1 with concentrated H_2SO_4 to measure SOC under heating in oil at 170-180 °C for 5 minutes (LY/T1237-1999; Bao, 2000). The detailed measuring procedure for SOC was conducted as follows:

1. Grinding the air-dried soils by mortaring them and then passing them through a 0.25 mm sieve.
2. Receiving 0.01-0.5 g (with accuracy of 0.0001g) sieved soil samples by using an analytical scale.

3. Transferring the soil into the test tube and then adding 5 ml 0.8 mol L^{-1} 1/6$K_2Cr_2O_7$ and 5 ml concentrated H_2SO_4.
4. Putting the test tube (with soil sample, $K_2Cr_2O_7$ and concentrated H_2SO_4) into an iron cage and then putting them into oil at 170-180 °C for 5 minutes.
5. When cooled down, washing the test tube carefully into a 250 ml filter flask using deionized water. Hereby keeping the volume in the filter flask at 60-70 ml until the solution is showing the colors crocus or canary.
6. Dropping 3-4 drops of "ferroin" indicator and titrating with $FeSO_4$ solution until the solution becomes brownish red. Recording volume of the consumed $FeSO_4$ solution.
7. Meanwhile, using silica sand as blank samples to measure the volume of the consumed $FeSO_4$ solution four times (using the average of the four repetitions).
8. Putting 10 ml 0.2 mol L^{-1} $K_2Cr_2O_7$ solution into a 100 ml filter flask, adding 2 ml concentrated H_2SO_4, dropping 3-4 drops of "ferroin" indicator and then titrating with $FeSO_4$ solution for the standardization of the $FeSO_4$ solution concentration. Repeating four times and using the average.

However, to measure LOC, the same steps were performed but with different concentrations and temperatures. We used 0.4 mol L^{-1} 1/6$K_2Cr_2O_7$ instead of 0.8 mol L^{-1} 1/6$K_2Cr_2O_7$ in step (3), concentrated H_2SO_4–H_2O (1:1) solution instead of concentrated H_2SO_4 in step (4), and 130-140 °C instead of 170-180 °C in step (4) (Yuan, 1963; Bao, 2000).

References:

Wu, J., Joergensen, R., Pommerening, B., Chaussod, R. & Brookes, P. Measurement of soil microbial biomass C by fumigation-extraction-an automated procedure. Soil Biology and Biochemistry 22, 1167-1169 (1990).

Hu Y-H, Sha L-Q, Blanchet FG, Zhang J-L, Tang Y, Lan G-Y and Cao M (2012). Dominant species and dispersal limitation regulate tree species distributions in a 20-ha plot in Xishuangbanna, southwest China. Oikos

121(6): 952-960.

Zhang Wanru, Yang Guangjin, Tu Xingnan, Zhang Ping. Determination of organic carbon in forest soil and calculation carbon-nitrogen ratio (LY/T1237-1999). Beijing: Forestry Research Institute of Chinese Academy of Forestry, 1999 (In Chinese).

Bao S. Soil and agricultural chemistry analysis (the third version). Beijing, China Agriculture Press, 2000: 31-35.

Yuan K (1963). Studies on the organo-mineral complex in soil. I.The oxidation stability of humus from different organo-mineral complexes in soil (Chinese version with English abstract). Acta Pedologica Sinica 11(3): 286-293.

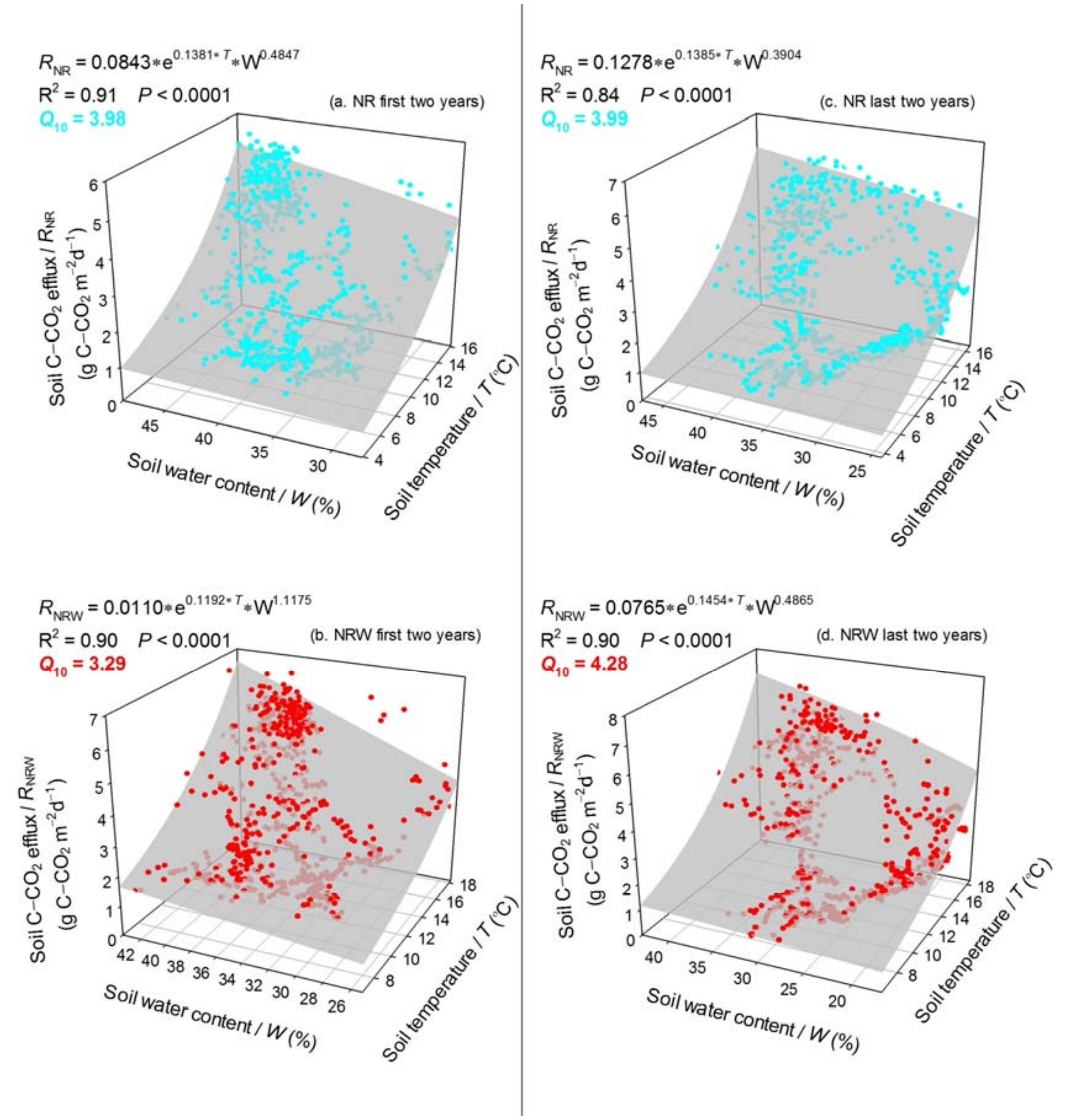

Fig. S3 Temperature sensitivities (Q_{10}) estimated from two-factor regression of soil carbon efflux with soil temperature and soil water content. The left part is the first two years data; the right part is the last two years data.

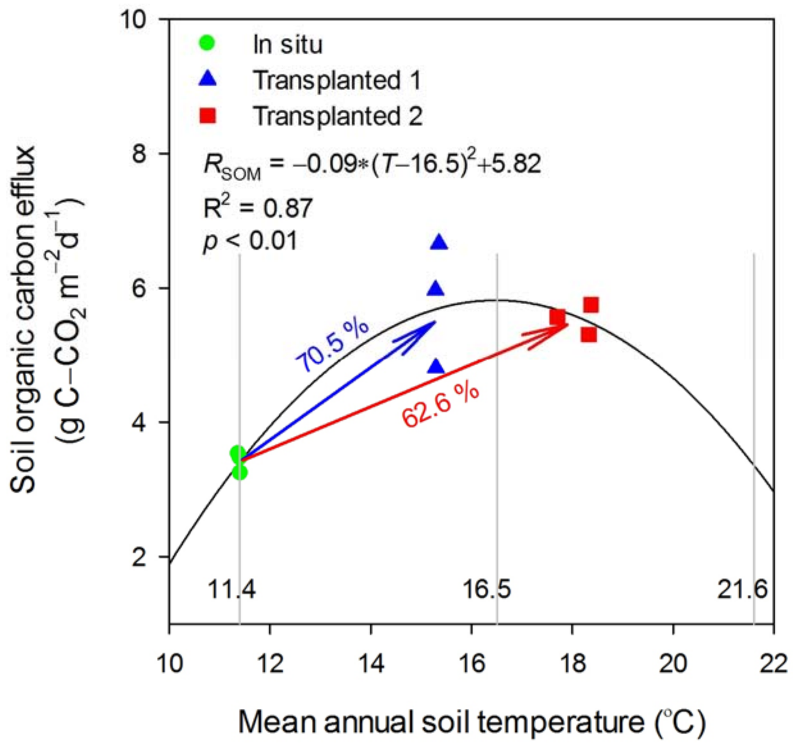

Fig. S4 Results of soil monolith transplantation experiments. Nine soil monoliths were selected in the beginning of January 2010. Three of them were transplanted to the middle elevation site (Transplanted 1), three were transplanted to the low elevation site (Transplanted 2), and three remained in situ. Because different tree species in different elevations have different litterfall, cover structures (i.e., a bamboo framework covered with 1-mm nylon mesh, 1 × 1 m) were established at a height of 1.2 m above the soil monoliths in three elevations to prevent new litter dropping. The experiment was established in the middle of January 2010, and measurement began from February 2010 to January 2012. Because of the variable rainfall and evaporation at different elevations, the two-factor regression model was used as a correction, as described in the data analysis section. Results show that the soil carbon efflux increased after soil was transplanted and exhibited a quadratic relationship with soil temperature.

Table S1 The Pearson correlation coefficient of soil carbon efflux (*R*) with soil temperature (*T*) and soil water content (*W*).

Treatment	*T*	*W*
CK	$r = 0.89, p < 0.001$	$r = 0.50, p < 0.001$
NR	$r = 0.90, p < 0.001$	$r = 0.45, p < 0.001$
NRW	$r = 0.90, p < 0.001$	$r = 0.50, p < 0.001$

Table S2 Estimation result of parameters from two-factor regression.

Treatments	Parameters	Coefficient	Std. Error	t value	*p*
	a	0.1137	0.0084	13.5229	<0.0001
CK	b	0.1491	0.0020	73.7164	<0.0001
	c	0.4468	0.0223	20.0622	<0.0001
	a	0.1561	0.0145	10.7484	<0.0001
NR	b	0.1419	0.0020	70.0780	<0.0001
	c	0.3134	0.0268	11.7036	<0.0001
	a	0.0863	0.0067	12.8577	<0.0001
NRW	b	0.1359	0.0019	72.0731	<0.0001
	c	0.4836	0.0232	20.8367	<0.0001
